# Acute kidney injury associated with rhabdomyolysis in a patient with COVID-19

**DOI:** 10.1590/2175-8239-JBN-2020-0170

**Published:** 2021-03-05

**Authors:** Viviane Schmitt Jahnke, José Antonio Tesser Poloni, Carla Andretta Moreira Neves, Camila Peter, Claudia Elizabeth Thompson, Liane Nanci Rotta

**Affiliations:** 1 Universidade do Vale do Rio dos Sinos, São Leopoldo, RS, Brasil.; 2 Hospital São Camilo, Esteio, RS, Brasil.; 3 Laboratório Exame, Novo Hamburgo, RS, Brasil.; 4 Universidade Federal de Ciências da Saúde de Porto Alegre, Programa de Pós-Graduação em Ciências da Saúde, Porto Alegre, RS, Brasil.

**Keywords:** Acute Kidney Injury, Rhabdomyolysis, Coronavirus Infections, Urinalysis, Lesão Renal Aguda, Rabdomiólise, Infecções por Coronavirus, Urinálise

## Abstract

Rhabdomyolysis is defined as the breakdown of skeletal muscle leading to the release of muscle contents into the extracellular fluid. Patients with rhabdomyolysis can be asymptomatic or have myalgia symptoms, weakness, myoglobinuria with dark urine, significant electrolyte imbalance, and acute kidney injury. Here we describe a case on acute kidney injury associated to rhabdomyolysis in a patient with COVID-19.

## Resumo

A rabdomiólise é definida como a lise da musculatura esquelética levando à liberação do conteúdo muscular para o fluido extracelular. Pacientes com rabdomiólise podem ser assintomáticos ou apresentar sintomas de mialgia, fraqueza, mioglobinúria com urina escura, desequilíbrio eletrolítico significativo e lesão renal aguda. Aqui descrevemos um caso de lesão renal aguda associada à rabdomiólise em um paciente com COVID-19.

## Introduction

Rhabdomyolysis is defined as the breakdown of skeletal muscle leading to the release of muscle contents into the extracellular fluid. Although there is no official consensus, it is usually defined by a serum creatine kinase (CK) >1.000 U/L (4-5 times the normal upper limit)^1^^,^ 2. Patients with rhabdomyolysis can be asymptomatic or have myalgia symptoms, weakness, myoglobinuria with dark urine, significant electrolyte imbalance, and acute kidney injury^2^.

### Structured presentation of the case

**Figure 1:**
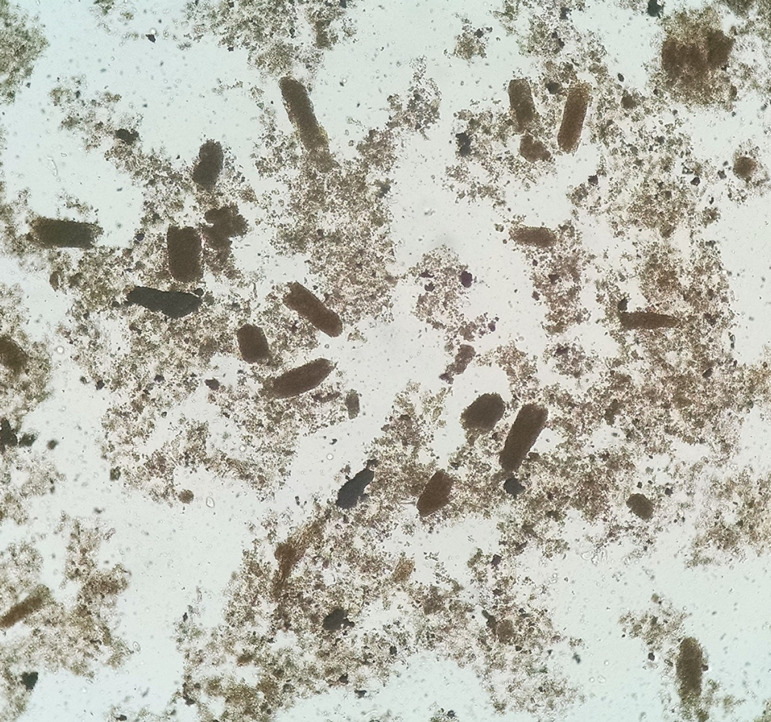
Fresh and unstained urine sediment showing several granular casts (muddy brown casts). Bright field microscopy. Original magnification 100x.

A 69-year-old female was admitted in the hospital on June 11, 2020, due to dyspnea and diarrhea. She lived in a nursing home with a previous history of systemic arterial hypertension, acute myocardial infarction, Alzheimer, and cardiopathy. At admission, the patient had little verbal response, torpor, tachydyspnea, shortness of breath, decreased vesicular murmur on the left side, slight snoring, and cold extremities; the blood pressure was 780/640 mmHg, body temperature of 36.2°C, heart rate of 79 BPM, respiratory rate of 26 breaths/min, and oxygen saturation of 88% on room air. A sample from the nasopharynx was collected with a swab and she tested positive for SARS-CoV-2 by RT-qPCR. Laboratory tests performed at admission revealed aspartate aminotransferase of 141 U/L (reference value - RV: <35U/L), alanine aminotransferase of 51 U/L (RV: <35U/L), lactate dehydrogenase of 521 U/L (RV: <248U/L), C reactive protein of 97.39 mg/L (RV: <5mg/L), impaired kidney function with a serum creatinine of 4.8 mg/dL, and an elevated level of serum CK of 4415 U/L (RV: <145U/L). The patient did not use nephrotoxins that could worsen kidney damage. On June 12, a urine sample (obtained using an indwelling urinary catheter) revealed the following results at dipstick test: specific gravity 1.010, pH 5.0, glucose 2+, and hemoglobin 2+; the other tests of the strip were negative. Urine sediment revealed 10 white blood cells (WBCs)/high power field (HPF), 6 red blood cells (RBCs)/HPF, 1 epithelial cell/HPF, 6 granular casts/low power field (LPF), and <1 waxy cast/LPF. The patient received fluid (isotonic intravenous fluids containing sodium bicarbonate to maintain urine output of 100-200 mL/h and urine pH >7.0), which improved kidney function with decreased serum creatinine (1.9 mg/dL) on June 13. However, CK level was still high and, despite treatment (diuretics, insulin, antipyretic, antibiotic, analgesic, antiemetic, and anxiolytic), the kidney function worsened, with serum creatinine reaching 4.2 mg/dL on June 16. Urinary microscopy performed on the same day revealed a clear picture of tubular injury with large amount of renal tubular epithelial cells (RTECs), RTECs casts, and granular casts (stained in brown color - muddy brown casts) (Figure 1). Due to the worsening of kidney function (Table 1), patient was transferred to a larger center. She died on June 18, 2020.

**Table 1:** Creatinine and Creatine kinase measurement over time.

Date	Creatinine (mg/dL)	Creatine kinase (U/L)
June, 11 2020	4.8	4415
June, 12 2020	4.4	Not performed
June, 12 2020	3.6	5014
June, 12 2020	2.1	3898
June, 13 2020	1.9	3689
June, 14 2020	1.9	1798
June, 15 2020	3.1	Not performed
June, 16 2020	4.2	Not performed
June, 17 2020	4.2	454

## Discussion

The most common viral causes of rhabdomyolysis are influenza A and B viruses followed by HIV and enteroviruses^3^. The report of other viruses as cause of rhabdomyolysis has been rare, including coronaviruses such as SARS-CoV-1^3^^,^ 4. Recently, a few adult cases have been reported. In a series of 1,099 patients with COVID-19 in China, 2 patients were diagnosed with rhabdomyolysis^5^. Another group in China analyzed the autopsies of 26 adult patients with COVID-19 and found pigmented casts associated with high CK levels in 3 patients^6^. The only case of COVID-19-associated rhabdomyolysis reported in the USA was of an 88-year-old male with subsequent development of mild acute kidney injury (different from the patient of this study) that resolved with intravenous fluid administration^7^. Borku Uysal et al. (2020) described a patient with COVID-19 pneumonia, with complaint of myalgia and a first diagnosis of mild rhabdomyolysis^8^.

Kidney injury results from a combination of ischemia due to renal vasoconstriction, direct tubular toxicity mediated by myoglobin-associated oxidative injury, tubular damage due to ischemia, and distal tubule obstruction due to the precipitation of the Tamm-Horsfall protein-myoglobin complex in addition to sloughed tubular cells forming cellular cast. As in acute kidney injury (AKI), endothelial dysfunction and local inflammation contribute to tissue damage and organ dysfunction^9^. After release into the extracellular fluid, myoglobin is filtered by glomeruli and is then reabsorbed in the proximal tubules by endocytosis. In tubular cells, myoglobin catabolism leads to free iron release, which becomes overwhelming during severe myoglobinuria. Free iron facilitates the generation of free radicals and oxidative stress damage, lipid peroxidation, and ultimately tubular cell injury and death, particularly in the setting of acidosis and aciduria^2^. A level of CK above 15.000-20.000 U/L indicates high risk for developing AKI, therefore a lower level, as observed in the current case, is in general one among several contributing risk factors for AKI.

The pathogenesis of different types of viral rhabdomyolysis has been in discussion, and involves the direct invasion of the muscle by virus, cytokine storm resulting in muscle damage, and muscle injury due to the circulating viral toxins. Chen et al. (2005) postulated that SARS-CoV-1-associated rhabdomyolysis was secondary to the cytokine storm rather than caused by direct viral invasion due to the presence of high inflammatory markers and the lack of viral particles on muscle biopsies^1^^,^ 4. There are no reports of muscle biopsies in patients with COVID-19-associated rhabdomyolysis, but similar to SARS, COVID-19 is also associated with a high level of inflammatory markers. This may indicate that rhabdomyolysis in COVID-19 could be cytokine-mediated^4^.

Rhabdomyolysis associated with SARS-CoV-2 infection can be an important contributing factor to the worsening of the clinical picture of patients with COVID-19, potentially leading to patient death. Suspected cases can be confirmed by the current gold-standard tests: CK and myoglobin (in urine and serum). CK can be measured in different isoenzyme forms depending on the damaged muscles. In rhabdomyolysis, CK-MM (found in skeletal muscle) is the predominant isoenzyme. There is no consensus on cutoff values for rhabdomyolysis diagnosis, but most studies have used values >1,000 U/L or 4-5 times the normal upper limit^2^^,^ 10. Plasma myoglobin is not as sensitive as CK for diagnosis due to its short half-life, resulting in false-negative tests^10^. With myoglobinuria, the urine dipstick test will be positive for blood in the absence of erythrocytes on microscopic examination, because the orthotoluidine portion of the dipstick turns blue in the presence of hemoglobin or myoglobin. When AKI is suspected, immediate assessment of AKI severity is mandatory by measuring serum K^+^, Ca^2+^, creatinine, urea, uric acid, and PO_4_^-^. Results will dictate the urgency and need of providing renal replacement therapy (RRT)^2^.

Several adult studies have evaluated predictive factors of rhabdomyolysis-induced AKI. Similar data in children is limited to smaller, retrospective studies. These studies suggest that the presence of dehydration, metabolic acidosis with aciduria, oliguria, massive muscle damage [as estimated by high serum myoglobin, CK, aspartate aminotransferase (AST) and lactate dehydrogenase (LD)], higher illness severity and systemic inflammatory response syndrome are predisposing factors for development of AKI with rhabdomyolysis^2^^,^ 11.

Treatment of rhabdomyolysis has not been well studied, and no specific treatment leads to a significant difference in outcome. Management is based on treating the underlying cause, preventing rhabdomyolysis in high-risk groups, using aggressive fluid resuscitation, administering diuretics, or alkalinization (based on poor evidence at best), and when required, RRT. However, in patients with severe or ongoing muscle injury, it may be reasonable to consider utilizing extracorporeal therapy to remove the inciting injurious agent, myoglobin, using a similar rationale to suspend a nephrotoxic medication (e.g., gentamicin) in the setting of severe nephrotoxic AKI^2^.
